# *In memoriam*: Celso-Ramon Garcia, M.D. (1922–2004), reproductive medicine visionary

**DOI:** 10.1186/1743-1050-2-2

**Published:** 2005-01-26

**Authors:** Jerome F Strauss, Luigi Mastroianni

**Affiliations:** 1 Center for Research on Reproduction and Women's Health, Department of Obstetrics and Gynecology, University of Pennsylvania, Philadelphia, Pennsylvania USA

## Abstract

This article traces the career of Celso-Ramon Garcia (1922–2004), noted physician, educator, and internationally renowned pioneer in the field of reproductive endocrinology. His work helped to formulate oral contraceptives used by millions of women throughout the world. Garcia's research collaborators included Gregory Pincus and John Rock, who together finalized the landmark clinical data needed to secure initial FDA approval for "the pill" in 1960. In addition to Garcia's monumental work in contraceptive endocrinology, his scholarly interests encompassed physiology of the menopause, minimally invasive reproductive surgery, as well as psychological aspects of infertility. Closely identified with the University of Pennsylvania, Garcia was instrumental in establishing the first formal clinical program in reproductive biology and influenced countless young scientists whose training he supervised and mentored. His distinguished career was emblematic of the best of the medical profession, characterized by compassion, intellect, and a sincere desire to help others. Our manuscript outlines Garcia's wide range of interests, acknowledges his superior fund of knowledge, and honors his humanitarian spirit – all of which contributed to an impressive legacy of medical discoveries. The impact of Prof. Garcia's work will continue to be felt for many years.

## Introduction

**Figure 1:**
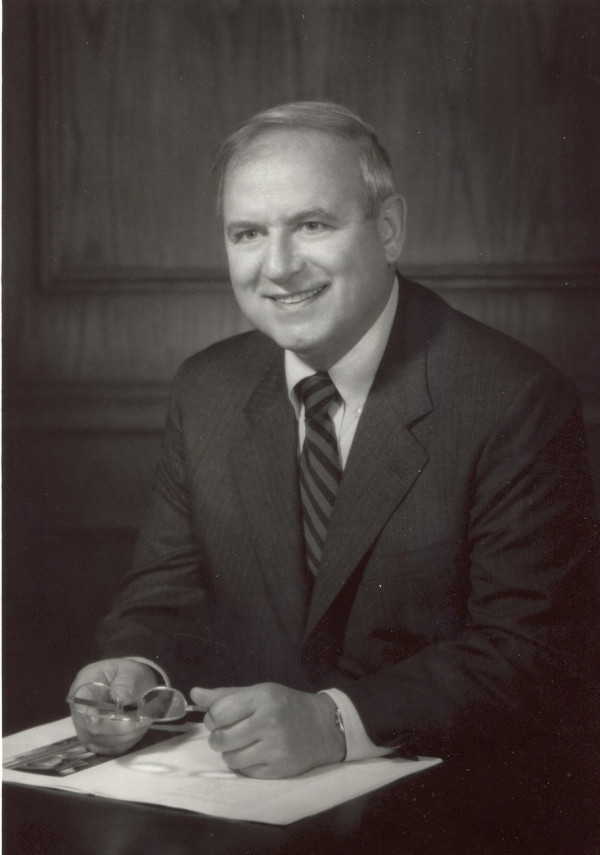
Celso-Ramon Garcia, M.D. (1922–2004)

Celso-Ramon Garcia (Figure 1), who departed this world in 2004 at the age of 82, was a remarkable human being who did much to shape the specialty of reproductive medicine as it is known today. His research yielded important discoveries that continue to influence the daily lives of millions of women and men around the globe. Reviewing his career and his accomplishments is like looking through a kaleidoscope – elements of greatness and the purest embodiment of the medical arts are brought together and with a small twist transformed into a new vision. Hard work and determination brought him from a relatively modest but academically-oriented Spanish immigrant family to medical training in community hospitals in Brooklyn, and then into the elite circles of East Coast academic medicine.

## Background

Garcia was raised speaking Spanish, but his English diction and elocution were so honed by his teachers in grammar and high school that hardly a trace of accent was left to be detected by the Boston Brahmans he would later encounter. Along the way his interests took seemingly improbable turns, yet there was a clear rationale that was beyond the imagination of the average physician. Indeed, his work presaged several important developments in the field of reproductive medicine. Garcia began his career with an interest in obstetrics, but ended up conducting clinical research on contraception that on more than one occasion was nominated for the Nobel Prize in Medicine or Physiology. He was revered as a meticulous abdominal infertility surgeon, yet he embraced the fledgling field of endoscopy and endoscopic surgery. While being celebrated for his surgical technique, he promoted research into the psychological aspects of infertility. Towards the end of his career he became interested in sexuality in the menopause, bringing his career through the full cycle from obstetrics, to family planning, to infertility to reproductive aging. His accomplishments in each of these spheres were well recognized by his peers who chose him President of the American Association of Planned Parenthood Physicians, Chairman of the Board of Directors of Planned Parenthood of America, Founding President of the Society of Reproductive Surgeons, and President of the American Society of Reproductive Medicine and bestowed upon him the Scientific Leadership Award from the Global Alliance for Women's Health, a non-governmental organization of the United Nations.

## Early years

Garcia's course to greatness was based on a diverse set of skills that he used creatively throughout his career. He majored in chemistry at Queens College where he revealed a remarkable aptitude for analysis of organic compounds. During his undergraduate studies, Garcia served as a teaching fellow in chemistry at New York University and authored two papers on qualitative and quantitative analysis. These are the earliest public manifestations of his passion for rigor and precision, which subsequently guided his clinical practice and his scholarship. After receiving his baccalaureate degree he entered medical school at the Long Island College of Medicine (now SUNY-Downstate) and graduated in 1945. He completed a rotating internship at the Norwegian Hospital in Brooklyn and then served two years in the U.S. Army Medical Corps in Fairbanks Alaska and then the Phoenixville General Army Hospital outside of Philadelphia. Garcia started a residency in pathology at Cumberland Hospital in Brooklyn where he was introduced to Sam Lubin, who became his friend and mentor. Lubin's work focused on uterine pathology and contractility and this sparked Garcia's interest in obstetrics and gynecology. With Lubin, Garcia would later publish papers on the effects of meperidine on labor.

## Further studies and research

After a year of pathology residency and a year as a research fellow, Garcia transferred to the obstetrics and gynecology program at Cumberland Hospital. With a pathologist's keen eye and an appreciation of tissue biology/healing, Garcia was well-equipped to excel in the art of reproductive surgery. In the operating rooms at Cumberland Hospital Garcia met Shirley Stoddard, the newly appointed Director of Surgical Nursing; she was to become his wife and lifelong companion. After completing residency, Garcia explored openings in academia but few opportunities were available. One exception was an advertisement for assistant professor of obstetrics and gynecology at the newly established medical school at University of Puerto Rico, for which Garcia applied and was accepted in 1953.

Garcia's research during residency, mentored by Lubin, had focused on obstetrical issues – something that he could have easily developed as a junior faculty member at the University of Puerto Rico. However, Garcia was about to embark on the first of several sharp turns that would lead him into seemingly improbable arenas. His arrival in Puerto Rico fortuitously coincided with the time for the first large scale clinical trials of the oral contraceptive pill. Within a year of Garcia's arrival, Gregory Pincus (director of the Worcester Foundation for Experimental Biology in Shrewsbury, Massachusetts) was in Puerto Rico looking for sites to conduct these trials. It was there that Pincus was first introduced to Garcia. Pincus had an uncanny ability to sense talent; he always surrounded himself with the brightest people. Pincus, undoubtedly impressed by Garcia's fund of knowledge in organic chemistry, training in pathology, and of course, the fact that he was bilingual in addition to his training in obstetrics and gynecology, promptly recruited Garcia to oversee his important research program in Puerto Rico.

Pincus would subsequently introduce Garcia to John Rock, who arranged for Garcia to be named Sydney Graves Fellow in infertility and gynecology at the Free Hospital for Women in Brookline, Massachusetts. There, Garcia continued to work on oral contraceptives but also pursued clinical work in infertility. Although the Graves fellowship no longer exists, contemporary reproductive medicine was developed by its incumbents. Rock had an interesting manner of selecting these fellows. He usually interviewed candidates over breakfast in his hotel room at the annual meeting of the American College of Obstetricians and Gynecologists. He could identify the smart ones within a minute, and had little patience for individuals who were not well-spoken. The diction and elocution drilling Garcia had endured and his insistence on precision brought him through with flying colors.

Garcia quickly became indispensable to Rock. Garcia moved to Boston, where he held assistant and instructor faculty posts at Harvard Medical School. During this time, Garcia also commuted to Puerto Rico to manage the oral contraceptive trials. Rock had a thriving infertility practice and Garcia did much of the surgery. Garcia's contributions to Rock's clinical practice were, however, underappreciated and when it came time for Garcia to be proposed for a full-time position at the Free Hospital for Women, the job did not materialize. This scenario is not unfamiliar to practitioners in our field, and it elicited the obvious reaction: Rock and Garcia pulled up stakes and took their hugely lucrative practice to the Faulkner Hospital in Boston. But after a short interval, the Rock/Garcia partnership was persuaded to return to the Free Hospital where an appropriate appointment was arranged for Garcia. Mrs. Stanley McCormick, a benefactor of remarkable vision who had provided initial funds to Pincus and colleagues at the Worcester Foundation to begin studies on oral contraceptive steroids, helped establish the Rock Reproduction Study Center at the Free Hospital campus, greatly facilitating research and training.

## The Worcester Program and Massachusetts General Hospital years

By 1960, Pincus had recruited Garcia to the Worcester Foundation as a senior scientist and director of the training program in Physiology of Reproduction. Sponsored by grants from the Ford Foundation and National Institutes of Health, this was the first training program of its kind. The discipline of reproductive biology as we know it today had its roots in the Worcester program. Although Garcia was appointed chief of the infertility clinic at Massachusetts General Hospital in 1962, he continued to visit Puerto Rico to perform follow-up examinations on women who had participated in the oral contraceptive trials in gratitude for their role and with genuine concern for potential untoward long-term effects. That was a personal mission, not a mandated component of the study protocol. This post-trial surveillance helped identify rare side effects that ultimately led to product reformulation and lowering of the steroid content of oral contraceptives. Garcia subsequently carried out groundbreaking studies on patient acceptance of oral contraceptives, recognizing that future developments in family planning must address the social and demographic aspects, including cultural differences in preferences for methods.

The technologies that gave men and women the ability to control their own reproduction through contraception and infertility treatments are arguably among the most important medical advances of the 20^th ^century. This is in good part Garcia's legacy [1-3]. While his career embraced the full spectrum of reproductive medicine and surgery, undoubtedly Garcia's most significant role was as co-developer of the first oral contraceptive approved by the U.S. Food and Drug Administration.

Important medical advances like "the pill" or IVF do not come without controversy, societal baggage, and rhetoric. The pill's creators fully understood these implications, and knew such peripheral distractions would take away from the significance of their work. Consequently, there was no phone call from Sweden despite several nominations to the Nobel committee. But it is best to look back on the history of the development of the oral contraceptive with awe and wonder. Indeed, the commercial availability of oral contraceptives was also important because it established a formal approval and regulatory framework for pharmacologic agents of an entirely new class: drugs designed to be used by healthy people for long periods of time. It provided a clear understanding of how post-marketing surveillance can lead to product reformulations that reduce side effects without reducing efficacy. It yielded an understanding of how sociology and demography must be incorporated into the development of pharmaceuticals and the practice of medicine.

## Garcia the peerless surgeon

Garcia maintained his connections with Rock and Rock's family of protégés, one of whom (L.M.) later lured him and other Rock/Pincus trainees, Edward Wallach and John V. Kelly, to the Department of Obstetrics and Gynecology at the University of Pennsylvania. Garcia spent the rest of his career at Penn developing the specialty of reproductive surgery, particularly microsurgical approaches. Here again came the totally un-expected. Garcia was at his best when confronted with the most challenging open abdominal procedures, which always ended with the pelvis in a pristine state, not a drop of blood, all surfaces meticulously re-peritonealized, no risk of inflammation or adhesion. Garcia's novel regimen of intraperitoneal corticoids and antihistamines (an early anti-adhesion therapy) was merely icing on the cake. The two-stage tuboplasty procedure was celebrated both locally and internationally. Yet this master of the open abdomen quickly recognized the potential of minimally invasive surgery, establishing one of the first endoscopic surgery programs in USA after a brief sabbatical with his colleague, Professor Kurt Semm in Kiel, Germany.

During his career, Garcia authored numerous textbook chapters on surgery of the ovaries, tubes and uterus that guided a generation of gynecologists in reconstructive surgery. He was also an early advocate of laser and electrosurgical methods, as well as microsurgical techniques designed to minimize tissue trauma. When Garcia's residents and fellows under-performed, he could not mask his displeasure, yet when they excelled he could not hide his joy. His concern for the outcomes of a rapidly evolving medical specialty led him to develop one of the first electronic infertility data registries [4].

In 1968, Garcia made yet another change in tack, developing an interest in psychological aspects of infertility. While this may have puzzled colleagues who knew Garcia only by reputation as a surgeon, those of us who worked with him knew well his holistic commitment to medicine and his devotion – unprecedented even in the current era – to his patients and their families. He had a profound understanding of the human frailties that emerge during the rigors of infertility treatment. One of us (J.F.S.) assisted Garcia in the care of an attorney's wife who underwent a two-stage tuboplasty. The husband demanded to be present during all medical examinations of his wife, he tape recorded all sessions, and refused to pay for Garcia's services, despite the fact that she conceived within three months after the hoods were removed from her reconstructed tubes. Garcia did not press the attorney for payment – he knew that the treatment course was exceptionally arduous with considerable post-surgery discomfort. When this same lawyer called eight months later asking if Garcia would serve as an expert witness in a plaintiff's medical malpractice case, Garcia thanked him for his call and politely said his schedule would not permit it, never mentioning the unpaid bill. He forgave, but he did not forget.

## Final years and retirement

Later in his career, Garcia and his colleagues undertook studies on the menopause that presaged the current interest in female sexual dysfunction. In retrospect, this was a logical extension of his long-standing interest in the social and psychological aspects first of contraception and family planning, and later of infertility. Garcia published on perimenopausal sexuality, and reported the first studies the relationship between sex steroid levels and sexual behavior. Garcia also recognized the need for multidisciplinary collaboration in the care of women, particularly in the post-reproductive years, and co-authored an early self-help manual for menopausal women [5]. The Women's Wellness Program he established at the Hospital of the University of Pennsylvania opened years ahead of similar facilities elsewhere.

Garcia gracefully retired from practice to devote time to his wife and family. This life transition, seamlessly executed, surprised many who were familiar with Garcia's intensity. But there should have been no surprise. Garcia demanded the highest technical standards from residents and fellows in the operating room, the same standards that he held for himself. He would not carry on if he could not perform to those standards, and he would not neglect his wife who needed increasing medical attention.

## Epilogue

Throughout his professional life, Celso-Ramon Garcia was a major force driving innovation in clinical care, and ultimately the evolution of the field of reproductive medicine. Let all who follow in the arena of reproductive endocrinology honor his contributions, appreciate the unique attributes that made him a great physician and educator, and gain insight from a truly remarkable career.

## Competing interests

The author(s) declare that they have no competing interests.

## Authors' contributions

JLS and LM contributed equally to this work and its revisions.
